# Phase I trial of dimethyl fumarate, temozolomide, and radiation therapy in glioblastoma

**DOI:** 10.1093/noajnl/vdz052

**Published:** 2020-01-24

**Authors:** Danielle Shafer, Mary Beth Tombes, Ellen Shrader, Alison Ryan, Dipankar Bandyopadhyay, Paul Dent, Mark Malkin

**Affiliations:** 1 Inova Schar Cancer Institute, Fairfax, Virginia; 2 Massey Cancer Center, Virginia Commonwealth University, Richmond, Virginia; 3 Department of Neurology, Massey Cancer Center, Virginia Commonwealth University, Richmond, Virginia

**Keywords:** dimethylfumurate, glioblastoma, radiation therapy, temozolomide

## Abstract

**Background:**

Dimethyl fumarate (DMF), an oral agent approved for the treatment of relapsing–remitting multiple sclerosis (RRMS), has promising preclinical activity against glioblastoma (GBM). This phase I study sought to determine the recommended phase 2 dose (RP2D) of DMF and evaluate its safety and toxicity when combined with standard concurrent radiotherapy (RT) and temozolomide (TMZ) followed by maintenance TMZ in patients with newly diagnosed GBM.

**Methods:**

Using a standard 3 + 3 dose-escalation design with 3 dose levels, patients received daily DMF with 60 Gy RT and concurrent TMZ 75 mg/m^2^ daily, followed by maintenance DMF (continuously) and TMZ 150–200 mg/m^2^ on days 1–5 of each 28-day cycle for up to 6 cycles. The maximum tolerated dose (MTD) was determined by evaluation of dose-limiting toxicity (DLT) during the first 6 weeks of therapy.

**Results:**

Twelve patients were treated at the 3 dose levels, and no DLTs were observed. There were no unexpected toxicities. The most common grade 3/4 treatment related adverse events (AEs) were lymphopenia (58%), decreased CD4 count (17%), and thrombocytopenia (17%). Four patients completed all planned treatment; seven patients had progression on treatment. One patient chose to withdraw from the study during maintenance. The median progression-free survival (PFS) for all patients was 8.7 months with no difference in PFS between those with stable disease or a partial response; median overall survival was 13.8 months.

**Conclusions:**

DMF may be safely combined with RT and TMZ in patients with newly diagnosed GBM. The RP2D for DMF is 240 mg three times daily.

**Importance of the Study**

We report the results of a phase I clinical trial evaluating the safety and tolerability of dimethylfumurate (DMF) when combined with standard concurrent radiotherapy (RT) and temozolomide (TMZ) in patients with newly diagnosed glioblastoma (GBM). In preclinical studies, DMF, an oral agent approved for relapsing–remitting multiple sclerosis, appears to downregulate expression of the proinflammatory cytokines interleukin-6 (IL-6) and tumor necrosis factor-alpha (TNF-α) in activated microglial cells, and is synergistic with TMZ and RT in human GBM cell lines. Here we present our findings of the combination. When combined with standard therapy, DMF was safe and well tolerated.

**Key Points**

- DMF, an oral dug used in MS, is synergistic with TMZ and radiation, in GBM cell lines.
- DMF can be safely combined with TMZ and radiation in newly diagnosed GBM patients.

Glioblastoma (GBM) is the most frequent and lethal primary brain tumor. Despite recent advances, the prognosis remains dismal.^1^ Attempts have been made to improve outcomes after maximum feasible resection. Given the infiltrating nature of GBM, even if gross total resection is accomplished, the addition of other treatment modalities is always necessary. The current standard of care includes maximal safe resection followed by combination radiotherapy (RT) and temozolomide (TMZ), and then maintenance TMZ for 6 months.^2^ Despite recent advances, the overall outcomes remain poor, with median overall survival (OS) of 14.6 months. The addition of Tumor Treating Fields to the “Stupp protocol” adds modestly to OS—20.9 versus 16.0 months.^3^

Dimethyl fumarate (DMF) is an oral agent approved for the treatment of relapsing–remitting multiple sclerosis (RRMS).^4,5^ The therapeutic mechanism of DMF in RRMS has not been fully elucidated, but DMF can suppress reactive immune cell function. In GBM, the GBM cells and the microglia play a reciprocal supporting role within the tumor microenvironment. Growth factors secreted by the initially transformed astrocyte cause activation of adjacent microglia; growth factors then secreted by the activated microglia further enhance transformed astrocyte growth and genomic stability. In theory, a drug such as DMF could suppress the activation of microglia by the transformed astrocytes which would reduce the growth and chemotherapy resistance of the GBM cells. In addition, DMF may also prevent lymphangiogenesis by suppressing endothelial cell growth and preventing capillary formation.^6^ Preclinical studies have identified that the expression of the proinflammatory cytokines interleukin-6 (IL-6) and tumor necrosis factor-alpha is decreased after activated microglial cells are exposed to DMF.^7^ IL-6 has been implicated in GBM cell invasion and angiogenesis. In glioma cells, DMF inhibits NF-ΚB signaling and blocks radiation-induced p65 phosphorylation. DMF reduces radiation-induced p65 phosphorylation. DMF also reduces radiation-induced activation of ERK1/2 and AKT and, as a single agent, activates p38 MAP kinase OR mitogen-activated protein kinase (MAPK). In vivo, DMF is rapidly metabolized to monomethyl fumarate (MMF). MMF appears to be synergistic with both radiation and TMZ in multiple human GBM cell lines. Furthermore, MMF does not interfere with the cytotoxic effects of TMZ in multiple human GBM cell lines. Based on preclinical studies, we initiated a phase I study to combine DMF with standard of care TMZ and RT to evaluate safety and tolerability in patients with newly diagnosed GBM.

## Materials and Methods

### Patient Selection

Patients greater than or equal to age 18 with a histopathologically proven diagnosis of GBM or gliosarcoma (WHO Grade IV) were eligible for the study. Other eligibility criteria included an Eastern Cooperative Oncology Group (ECOG) performance status of ≤2 and adequate bone marrow function as defined by absolute neutrophil count ≥1500/mm^3^, platelet count ≥100,000/mm^3^, and hemoglobin ≥10 g/dL. Additionally, adequate organ function was required as defined by creatinine ≤ 1.5 × upper limit of normal (ULN) or calculated or actual creatinine clearance > 45 mL/minutes, total bilirubin ≤ 1.5 × ULN and Aspartate Aminotransferase (AST)/Alanine Aminotransferase (ALT) ≤3 × ULN. Patients were required to begin therapy within 3–6 weeks of most recent brain tumor surgery, biopsy, or resection; patients were required to recover from surgery or biopsy before registration and remain on stable or decreasing dose of steroids in the week prior to registration. Exclusion criteria included clinically significant cardiac disease or pregnant or lactating women. Patients with prior invasive malignancy were also excluded unless disease free for ≥3 years. In addition, patients with recurrent malignant gliomas or prior chemotherapy or RT for the diagnosis of GBM or cancers of head and neck or metastases below the tentorium or beyond the cranial vault were also excluded. Informed consent was obtained from the patients prior to enrollment. The protocol was approved by the Institutional Review Board at Virginia Commonwealth University and registered with clinicaltrials.gov (NCT02337426).

### Objectives

The objective of this phase I study was to determine the recommended phase 2 dose (RP2D) of DMF when combined with standard concurrent TMZ and RT in patients with newly diagnosed GBM. Secondary objectives were to determine the safety, tolerability, and toxicity of the combination and to assess preliminary efficacy results.

### Study Design

This was a phase I single-arm, single-institution, dose-escalation study of DMF in combination with standard chemoradiotherapy in adult patients with newly diagnosed GBM. DMF was administered orally continuously from day 1 of RT until completion of maintenance TMZ. RT was considered standard for the treatment of GBM. For both intensity-modulated radiation therapy and 3D-conformal therapy plans, one treatment of 2.0 Gy was given daily for 5 days per week for a total of 60.0 Gy over 6 weeks. Per routine clinical care, TMZ was administered at a dosage of 75 mg/m^2^ daily for 42 days concurrent with RT. Maintenance TMZ was administered for six cycles at a dosage of 150–200 mg/m^2^ daily on days 1–5 of every 28-day cycle. There were 3 dose levels of DMF: 120 mg twice daily, 240 mg twice daily, and 240 mg three times daily. The Federal Drug Administration (FDA)-approved dose of DMF for RRMS is 240 mg twice daily. As per the DMF package insert, there was a run-in period for patients in dose levels 2 and 3 with DMF 120 mg twice daily on days 1–7. The phase I study followed a standard 3 + 3 dose-escalation design with three patients enrolled in a dose level cohort. If the first three patients tolerated the first 6 weeks of study treatment without experiencing a dose-limiting toxicity (DLT), the next dose level was permitted to enroll. The maximum tolerated dose (MTD) was defined as the highest dose level at which <33% of patients in a cohort experienced a treatment-related DLT. Determination of the RP2D required six DLT-evaluable patients.

### Assessments

Response was assessed via RANO criteria prior to start of maintenance therapy and then after cycles 2, 4, and 6 (study completion).^8^ Adverse events (AEs) were graded according to National Cancer Institute Common Terminology Criteria for Adverse Events (NCI-CTCAE) version 4.0. Patients were evaluated for AEs from first dose of study treatment until 30 days after discontinuation of treatment. Patients were then followed for survival and new treatment initiation.

## Results

### Patient Characteristics

A total of 12 patients with primary GBM were enrolled from April 2015 through September 2016. Three patients were treated at dose levels 1 and 2; six patients were treated at dose level 3. The baseline characteristics are summarized in Table 1. The median age was 68 years (range 40–82).

**Table 1.** Patient characteristics

	All (*n* = 12)
Median age, year (range)	68 (40–82)
ECOG PS	
0	3
1	7
2	2
Gender	
Male	8
Female	4
Race	
White	10
Black	1
American Indian or Alaska Native	1
Diagnosis	
Glioblastoma	11
Gliosarcoma	1
Surgery	
Biopsy	1
Partial resection	6
Gross total resection	5

### Toxicity

No DLTs were identified. Grade 3/4 AEs possibly, probably, or definitely related to treatment are outlined in Table 2. Toxicities were expected, were primarily hematologic, and were chiefly related to TMZ. Grade 3/4 AEs of lymphopenia and decreased CD4 lymphocytes were also attributed to DMF as it may cause lymphopenia. Despite the lymphopenia, no serious infections, including *Pneumocystis jiroveci*, were observed. The only non-hematologic treatment related grade 3/4 AE was a single grade 3 hemorrhoid event attributed to TMZ. The most common non-hematologic toxicities attributed to DMF were ALT elevation (50%), AST elevation (33%), proteinuria (25%), nausea (17%), diarrhea (17%), and flushing (17%); all were grade 1. No patients discontinued study for toxicity; one patient electively withdrew from treatment during maintenance.

**Table 2.** Grade 3/4 treatment related adverse events

	Grade 3	Grade 4
Hematologic, *n* (%)		
CD4 decreased	2 (17%)	1 (8%)
Lymphocyte count decreased	7 (58%)	2 (17%)
Neutrophil count decreased	0	1 (8%)
Platelet count decreased	2 (17%)	0
White blood cell decreased	1 (8%)	0
Non-hematologic, *n* (%)		
Hemorrhoids	1 (8%)	0

*Note*: Percentages are all grades per patient.

### Response

All 12 patients were evaluable for response. All 12 patients completed chemoradiotherapy and initiated the maintenance phase for 3–6 cycles. Best response and reason for discontinuation are outlined in Table 3. Four patients completed all planned treatment; seven patients had progression during treatment. The median progression-free survival (PFS) for all patients was 8.7 months. There was no difference in PFS between those with stable disease (SD) and a partial response (PR). The median OS for all patients was 13.8 months with no difference in those with SD or PR. For the six patients treated at the highest dose level, median PFS was 11.8 months, and median OS was 14.5 months.

**Table 3.** Best response and reason for discontinuation

#	Cohort level	Best response	Reason for discontinuation
1	1	SD	Disease progression
2	1	PR	Disease progression
3	1	SD	Treatment completed
4	2	SD	Disease progression
5	2	SD	Disease progression
6	2	SD	Disease progression
7	3	SD	Disease progression
8	3	SD	Treatment completed
9	3	PR	Treatment completed
10	3	PR	Disease progression
11	3	SD	Elective withdrawal during maintenance
12	3	PR	Treatment completed

PR, partial response; SD, stable disease.

## Discussion

This phase I dose-escalation study evaluated oral DMF in combination with standard chemoradiotherapy for patients with newly diagnosed GBM. To our knowledge, this is the first clinical trial to test DMF as anticancer treatment for GBM. Oral daily administration of DMF in combination with RT and TMZ demonstrated an acceptable safety profile. No DLTs were observed during the study. While the MTD in our study was 240 mg three times daily, it is notable that the FDA-approved dose for RRMS is 240 mg twice daily. In the clinical trials leading to DMF approval for relapsing form of multiple sclerosis (MS), there was no difference in primary outcomes between the 240 mg twice daily arm and 240 mg three times daily arm. While it may be difficult to justify exceeding the FDA-approved dose without additional testing in the GBM population, we cannot definitively state that twice daily and three times daily dosing have equivalent efficacy in the GBM population. A larger study would be necessary to appropriately assess dosing efficacy.

In the RRMS studies leading to DMF approval, AEs with incidence greater than 10% included flushing, abdominal pain, diarrhea, and nausea.^4,5^ We did not see similar toxicity in our population. It is notable that patients on our study had ready access to prophylactic ondansetron anti-emetic therapy concomitant with TMZ. DMF has also been described to cause lymphopenia. In the RRMS placebo-controlled trials, lymphocyte counts decreased by approximately 30% during the first year of treatment; 6% had grade 2 lymphopenia. Despite this hematologic toxicity observed in RRMS, there was no increase in serious infections in the DMF arms as compared to the placebo arm. In our small study, we did not see any serious infections related to the DMF and TMZ combination. Without a larger study comparing standard Stupp therapy to a DMF–Stupp combination, it would be difficult to assess the contribution of DMF to lymphopenia as TMZ has greater hematologic toxicity, including lymphopenia, than DMF.

The current study was not powered to detect efficacy. In general, the PFS and OS observed in the limited number of patients on our study were within range of reported results from prior newly diagnosed GBM studies. A phase 2 study would be necessary to detect if the PFS and OS were meaningful and merit further investigation. A decision was made by the manufacturer to stop further development of DMF in the GBM population. In summary, we report that the addition of DMF to standard therapy with RT and TMZ in patients newly diagnosed GBM appeared to be safe without unexpected toxicity.
